# William Mitchell

**Published:** 1914-07-15

**Authors:** 


### OBITUARY.

William Mitchell. Dr. Wm. Mitchell of London, England, died June 9th, 1914, after an extended illness. Dr. Mitchell's early home was in Delaware, Ohio. He studied dentistry in the University of Michigan from which he was graduated in 1878. He soon after went to London, England, where he established one of the best known American practices. He retired from practice six or seven years ago because of ill health. He was an energetic believer in American dentistry and strenuously defended it when ever occasion required. He did much to secure recognition for our dental degrees in England, especially for American schools of the best class. He did much to assist Americans in establishing themselves in practice in England. He was one of the strongest supporters of the American Dental Society of Europe and contributed many papers to dental literature through that society.

When in college he became greatly interested in Comparative Dental Anatomy through the teachings of Prof. Corydon L. Ford, and he made a large and choice collection of animal crania from all parts of the world. When he retired from practice he sent his entire collection to the Dental Department of the University of Michigan. When combined with the collection of Prof. Ford, it makes what is probably the best collection of dental crania in the country.

Dr. Mitchell was a good business man and from his practice and judicious investments accumulated a considerable estate. He never married and leaves a brother Dr. Louis J. Mitchell, who is practicing dentistry at 90 Park Str., Grosvenor Square, West London.

Dr. Mitchell had many friends in America and he was a frequent visitor to this country until recent years. Those whose pleasure it was to have known him will be deeply grieved to learn of his death. He was a man of strong character and high professional ideals. He loved his profession and was jealous of its good name. He did much to establish a comradship among Americans practicing in Europe and no American dentist in Europe was better known or had a greater influence. He was not a scientific worker but a most skillful practitioner, and contributed to the technical literature of the profession many papers of value, most of which were published in our American Dental Journals.

